# SARS-CoV-2: Should We Be Concerned about the Nervous System?

**DOI:** 10.4269/ajtmh.20-0447

**Published:** 2020-07-17

**Authors:** Marcus Tulius T. Silva, Marco Lima, Abelardo Q.-C. Araujo

**Affiliations:** 1 Brazilian Ministry of Health, Evandro Chagas National Institute of Infectious Diseases (INI), Fundação Oswaldo Cruz, Rio de Janeiro, Brazil;; 2 Niteroi’s Hospital Complex, Rio de Janeiro, Brazil;; 3 The Federal University of Rio de Janeiro Medical School (UFRJ), Rio de Janeiro, Brazil

## Abstract

The COVID-19 pandemic has proved to be an enormous challenge to the health of the world population with tremendous consequences for the world economy. New knowledge about COVID-19 is being acquired continuously. Although the main manifestation of COVID-19 is SARS, dysfunction in other organs has been described in the last months. Neurological aspects of COVID-19 are still an underreported subject. However, a plethora of previous studies has shown that human CoVs might be neurotropic, neuroinvasive, and neurovirulent, highlighting the importance of this knowledge by physicians. Besides, several neurological manifestations had been described as complications of two other previous outbreaks of CoV diseases (SARS ad Middle East respiratory syndrome). Therefore, we should be watchful, searching for early evidence of neurological insults and promoting clinical protocols to investigate them. Our objectives are to review the potential neuropathogenesis of this new CoV and the neurological profile of COVID-19 patients described so far.

## INTRODUCTION

The world has been facing a pandemic for less than 6 months that has already resulted in thousands of deaths and is paralyzing the world economy. In December 2019, severe pneumonia cases of unknown origin were seen in Wuhan, China.^1,2^ The pathogen was rapidly identified as a novel enveloped RNA β-CoV, named SARS-CoV-2. SARS-CoV-2 quickly spread to other parts of China and later to all continents. Thenceforth, in March 2020, the WHO declared COVID-19 a new pandemic.

Several neurological manifestations were described as complications of two other previous outbreaks of CoV diseases, namely, SARS and the Middle East respiratory syndrome (MERS).^3–7^ Furthermore, recent clinical observations had stressed the possibility of neurological diseases also in the context of COVID-19.^8–11^ Therefore, we should be vigilant, searching for early evidence of neurological insults and promoting clinical protocols to investigate them. For instance, considering that encephalitis is associated with high mortality and morbidity, early diagnosis and management may contribute to better outcomes. Several clinical and laboratory studies have shown that human coronaviruses (HCoVs) might be neurotropic, neuroinvasive, and neurovirulent.^12–15^ The objectives of this article are to review the potential neuropathogenesis of this new CoV and the neurological profile of COVID-19 patients described worldwide.

## RESPIRATORY VIRUSES AND THE NERVOUS SYSTEM

Viral respiratory infections in humans are generally secondary to the human respiratory syncytial virus, influenza virus, HCoV, measles virus, rhinovirus, adenovirus, and human metapneumovirus.^16^ Transmission of these agents occurs mainly by contact with fomites or suspension droplets.^17^ All these viruses can produce bronchiolitis and pneumonia, being responsible for a large number of hospitalizations every winter season.^16,18,19^ In some cases, central nervous system (CNS) diseases can also be seen. It is well known that viral respiratory infection can result in several neurological disorders such as seizures, status epilepticus, encephalopathy, and encephalitis (Table 1).

**Table 1:** Neurological complications and principal laboratory findings associated with viral respiratory infection

Respiratory virus	Clinical signs	Laboratory observations
Human respiratory syncytial virus (human *Orthopneumovirus*)	Febrile seizure, convulsion, ataxia, status epilepticus, meningoencephalitis, cerebellitis, encephalopathy, and encephalitis	Viral antibodies in the CSF, viral RNA in the CSF (serogroups A and B). Elevated IL-6, IL-8, CCL2, and CCL4 in the CSF. Low levels of TNF-α in the CSF. Elevated IL-6 and BDNF in the CSF correlate with brain damage
Influenza	Febrile or afebrile seizures, myelitis, meningitis, encephalitis, Guillain–Barre syndrome, acute necrotizing encephalopathy, depression, neuritis, altered state of consciousness, delirium, and abnormal behavior	Pandemic H1N1 isolated from brain postmortem. Viral material in the CSF from patients (H1N1 and H3N2)
CoV	Febrile seizures, seizure, loss of consciousness, encephalomyelitis, and encephalitis	Viral detection in brain postmortem, from patients with multiple sclerosis (HCoV-229E and HCoV-OC43). Detection of SARS-CoV and HCoV-OC43 in the CSF
Human *metapneumovirus*	Febrile seizures, encephalopathy encephalitis, and status epilepticus	Viral RNA in brain postmortem. Viral RNA in the CSF from a patient

IL = interleukin; TNF = tumor necrosis factor; BDNF = brain-derived neurotrophic factor; H1N1 = influenza A subtypes H1N1; H3N2 = influenza A subtypes H3N2; HCoV = human coronaviruses. Adapted from Bohmwald et al.^72^

Respiratory viruses, in general, can invade the CNS through three main routes: the hematogenous route, through the infection of the endothelium or by transendothelial mechanisms; the “Trojan horse” mechanism, by which viruses in the bloodstream infect leukocytes that can transmigrate across the permeable blood–brain barrier; and through the olfactory nerves by axonal transport via olfactory neurons.

This last example is an elegant mechanism to access the CNS for a virus that enters the body intranasally, such as most of the respiratory viruses highlighted before.^20,21^ Furthermore, it is tempting to associate this route with anosmia, a frequent symptom of COVID-19.^22,23^ However, in experimental work, the probable route for brain infection in macaques was the hematogenous one.^24^ CoV RNA and CoV antigen were detected in nonhuman primates’ brain after intranasal, intraocular, or intravenous inoculation of murine CoV JHM OMp1. In this animal model, both the lack of detection of virus products in the trigeminal ganglia or olfactory bulbs and the presence of viral antigen in vessels and perivascular regions suggest that CoV entered the CNS through vascular endothelium.

## OVERVIEW OF COVS

CoVs are widespread and infect different species, generally causing mild, uncomplicated respiratory and enteric diseases. Usually, they infect the upper respiratory tract, being mainly associated with the common cold. However, in some patients, they can reach the lower respiratory tract, causing interstitial pneumonia, exacerbations of asthma, respiratory distress syndrome, or even SARS.^25^

CoVs are enveloped positive-sense RNA viruses characterized by club-like spikes that project from their surface, an unusually large RNA genome, and a unique replication strategy. CoVs are classified into four different groups; α-, β-, γ-, and Δ-CoV.^26^ SARS-CoV-2 is an RNA β-CoV with a characteristic crown-shaped appearance, grouped within the family Coronaviridae, order Nidovirales.

SARS-CoV-2 shares significant genetic homology with SARS-CoV, a virus associated with the pandemic of SARS that occurred in 2003. Similar to SARS-CoV, angiotensin-converting enzyme 2 (ACE2), an enzyme that physiologically counters renin–angiotensin–aldosterone system activation, is the functional receptor to SARS-CoV-2.^27^ It is known that the ACE2 receptor is also expressed in the brain.^28^

Several HCoVs are pathogenic to humans, such as HCoV-OC43, HCoV-229E, MERS-CoV, and SARS-CoV, all of them having different genotypes.^29–31^ Neurotropic and neuroinvasive abilities of HCoV have been described both in animals and humans, and is implicated in conditions such as multiple sclerosis and encephalomyelitis.^5,18^ Interestingly, the first detection of HCoV in the human brain was made at autopsy cases of multiple sclerosis in the early eighties.^32^ More recently, Arbour et al.^13^ detected the presence of the HCoV-OC43 in brain parenchyma samples of 35.9% patients with multiple sclerosis, compared with 13.7% of controls. Also, murine hepatitis virus, another CoV, has been linked to chronic inflammation and demyelination of the CNS in animal models.^33^ The viral glycoprotein S (spike) has an essential role for the neurovirulence, especially for the HCoV-OC4.^34^

### SARS-CoV.

SARS was a novel zoonotic infectious disorder associated with SARS-CoV. It was first diagnosed in China, in November 2002. The comparison of SARS-CoV sequences isolated from civets and patients supported the concept of transmission from these animals to humans.^35,36^ Phylogenetic analysis showed that SARS-CoV was not a novel CoV, but a branch of the β-CoV.^37–39^

Typically, SARS patients exhibited a triphasic pattern of disease, initially presenting with fever, a nonproductive cough, sore throat, and myalgia.^40^ Generally, dyspnea does not become a prominent feature until the second week of illness. In the second phase, dyspnea and hypoxia with fever become more prominent. Some patients progress to acute respiratory distress by the third week, often requiring mechanical ventilation. The severity of the disease was correlated with increasing age, and the mortality can reach 50% for patients older than 60 years.^40,41^

During the SARS-CoV outbreak, several neurological diseases were reported. Peripheral nervous system manifestations associated with SARS were described in four patients, including both axonal polyneuropathy and myopathy.^4^ The neuromuscular disorders developed approximately 3 weeks after the onset of SARS, and the prognosis was good. Interestingly, olfactory neuropathy was described during the SARS outbreak.^42^ This finding is relevant because, nowadays, many patients with SARS-CoV-2 infection have reported anosmia.^23^

A neuroinvasive behavior of SARS-CoV could be found in some other reports. SARS-CoV RNA was present in both the serum and cerebrospinal fluid (CSF) from a patient with status epilepticus and SARS during the 2003 outbreak.^43^ In another report, SARS-CoV RNA was recovered from a CSF sample of an infected patient admitted because of generalized seizures.^5^ In addition, a 39-year-old patient died of SARS after a severe chronic progressive viral cerebritis.^44^ Neuropathological examination showed gliocyte hyperplasia, neuron denaturation, and necrosis coupled with striated encephalomalacia. Neuroinvasion by SARS-CoV was confirmed by the typical viral morphology observed under electron microscopy, by genetic identification, and by the detection of the viral antigen (N protein) in the brain.

In a neuropathological study of patients with SARS, performed during the 2003 Chinese outbreak, sequences of SARS-CoV genome were detected in all brain samples, but in none of the control cases.^45^ The authors stressed that the infection of neurons occurred in selected areas such as the hypothalamus and cortex. The degeneration seen in these cases was probably secondary to neuronal hypoxia/ischemia.

It is hypothesized that the infection of neurons may explain a higher than usual percentage of neurological and psychological abnormalities observed in patients with late-stage SARS.^46^ Therefore, those neuropsychiatric symptoms could not be merely attributed to negative social pressure during the epidemic. Interestingly, it is not uncommon for survivors of SARS to report neuropsychiatric symptoms, such as lethargy, malaise, orthostatic dizziness, apathy, depression, and anxiety. Recently, studies have linked neuroendocrine aberrations to some neuropsychiatric conditions. Dysfunction in the hypothalamic–pituitary–adrenal axis was reported in survivors of SARS by Leow et al.^47^ In a cohort of 61 SARS patients, 39.3% had a central hypocortisolism. The authors speculated that either a hypophysitis or a direct viral hypothalamic infection could be the cause of these extrapulmonary symptoms.

All these clinical, laboratory, and neuropathological evidence suggest that CNS infection is possible in patients with SARS. Most HCoVs share similar viral structures and mechanisms of infection and have documented neurotropism. Therefore, infectious mechanisms previously found in other HCoVs may also apply to SARS-CoV-2.^48,49^ Taking these observations into account, a proactive search for neurological symptoms and signs could elucidate if the same occurs in SARS-CoV-2 infection.

### SARS-CoV-2.

SARS-CoV-2 is a new virus that shares almost 80% genomic homology with SARS-CoV. However, the highest level of similarity is with a horseshoe bat CoV.^50^ Therefore, it is believed that SARS-CoV-2 is a recombinant virus, transferred from bats to human hosts via an intermediate host.^51^ Because it is an RNA virus with an RNA-dependent RNA polymerase–based replication, mutation and recombination are not uncommon events.

SARS-CoV-2 has been recently associated with severe interstitial pneumonia, called COVID-19.^1,2^ COVID-19 is an acute viral pneumonia, potentially lethal in many cases. Characteristics of patients with severe evolution are the rapid progression to respiratory failure; it is estimated that among those with respiratory difficulties, 50% have to be admitted to intensive care units, and, of these, 46–65% worsen in a short period and may die in a few days.

Although SARS-CoV and SARS-CoV-2 use the same receptor to enter human cells, the ACE2 binding affinity of the SARS-CoV-2 spike protein is 10- to 20-fold higher than that of the SARS-CoV spike protein.^52^ Once within the CNS environment, its interaction with ACE2 receptors expressed in neurons can initiate a cycle of viral budding accompanied by neuronal damage, without substantial inflammation, as seen in the past in cases of SARS-CoV.^53^ It is also important to mention that long before the neuronal damages occur, endothelial tears in cerebral capillaries, followed by bleeding within the cerebral tissue, can have fatal consequences in patients with COVID-19.^54^

Experimental studies with HCoV and other viruses have shown the presence of viral particles in the brain, especially in the brainstem. Viral antigens of influenza and pseudorabies virus have been detected in the nucleus of the solitary tract and the nucleus ambiguous.^55,56^ The nucleus of the solitary tract receives sensory information from the mechanoreceptors and chemoreceptors of the lung and upper and lower airways, whereas the efferent fibers from the nucleus ambiguous and the nucleus of the solitary tract provide innervation to airway smooth muscle, glands, and blood vessels. Such neuroanatomical interconnections suggest that the death of many infected animals and even patients may be due to a dysfunction of the cardiorespiratory center in the brainstem.^53,57^ Likewise, experimental studies using transgenic mice have demonstrated brainstem infection by SARS-CoV and MERS-CoV.^53,57,58^ Whether the neuroinvasion of SARS-CoV-2 has a role in the development of respiratory failure in COVID-19 patients is still a matter of speculation. This information is essential for the prevention and better treatment of SARS-CoV-2–induced respiratory failure.

Several reports discussed neurological complications of COVID-19. In a clinical series, Giacomelli et al.^23^ described olfactory and taste disorders in 20 of 59 SARS-CoV-2–infected individuals. Lechien et al.^59^ studied 417 patients and found olfactory dysfunction in 85.6% and gustatory dysfunction in 88%. In 11.8% of patients, smell loss was the first symptom of COVID-19. Because the ACE2 receptor is widely expressed on the epithelial cells of the mucosa of the oral cavity and SARS-CoV exhibits a transneural penetration into the olfactory bulb, the pathogenetic mechanism of taste and olfactory disorders in SARS-CoV-2 infection could be justified.^53,60^

A case of a 56-year-old patient with COVID-19 and encephalitis was recently described; CSF tested positive for SARS-CoV-2 by gene sequencing.^61^ In another report, a woman with COVID-19 and acute necrotizing encephalopathy (ANE) was described.^9^ Acute necrotizing encephalopathy is a rare and potentially severe neurological complication of some viral infections, such as influenza. It has been associated with an exaggerated inflammatory response in the CNS named cytokine storm, responsible for a blood–brain barrier breakdown. Characteristic imaging features of ANE include symmetrical, multifocal lesions with invariable thalamic involvement. Lesions appear hypodense on computador tomography images, and magnetic resonance imaging (MRI) demonstrates T2/fluid attenuation inversion recovery hyperintense signal with internal hemorrhages. As in other severe viral infections, COVID-19 has been associated with cytokine storms.^62^

A case of Guillain–Barre syndrome (GBS) was recently published by Zhao et al.^11^ The authors described a 61-year-old woman infected by SARS-CoV-2 who developed acute weakness in both legs and severe fatigue, progressing within 1 day. Although it is not possible to exclude an epiphenomenon between SARS-CoV-2 infection and GBS, considering the temporal association, the authors speculate that SARS-CoV-2 might have been responsible for the development of GBS in this patient. In this report, the disease followed a parainfectious profile, instead of the classic postinfectious pattern, reported in GBS associated with other pathogens. More recently, Toscano et al.^63^ described a clinical series of five patients with GBS in Italy. The interval between the onset of symptoms of COVID-19 and the first neurological symptoms ranged from 5 to 10 days. CSF samples of all patients were negative to SARS-CoV-2. In three patients, the findings were consistent with an axonal variant of GBS and with a demyelinating process in two. All patients were treated with intravenous immune globulin.

In addition, two patients with SARS-CoV-2 infection were reported with Miller–Fisher syndrome and polyneuritis cranialis, respectively.^64^ In the first patient, vertical diplopia, perioral paresthesia, and gait ataxia were noted 7 days following COVID-19 symptoms (fever, anosmia, ageusia, low back pain, and malaise). This patient was treated with intravenous immune globulin, with the resolution of the neurological symptoms, except for anosmia and ageusia. Goh et al.^65^ recently described a 27-year-old man who developed isolated peripheral facial palsy on the sixth day of SARS-CoV-2 infection.

Mao et al.^8^ have published a clinical series on neurological aspects of COVID-19. Among 214 COVID-19 patients, 37% had some neurological manifestation, with nearly 50% having severe COVID-19 disease (Table 2). The authors drew attention to the fact that in patients with severe infection, the neurological involvement was more frequent, and included acute cerebrovascular diseases, disturbances of consciousness, and skeletal muscle injury. In this seminal series of cases, the nervous system involvement was associated with a poorer prognosis. The authors described mental status alterations in 15% of severe cases and nonspecific symptoms, including headache and dizziness, in nearly 20%. The authors referred to another common finding: “skeletal muscle injury” (creatine kinase > 200 IU/mL) that was seen in approximately 20% of severe cases. Unfortunately, the authors did not describe whether there were clinical manifestations suggesting myositis or myopathy, or even signs of acute motor neuron injury.

**Table 2:** Neurological manifestations in 214 hospitalized COVID-19 patients^8^

	Total (*n* = 214)	Severe COVID-19 (*n* = 88)	Non-severe COVID-19 (*n* = 126)	*P*-value
Nervous system symptoms, *n* (%)				
Any	78 (36.4)	40 (45.5)	38 (30.2)	< 0.05
Central nervous system	53 (24.8)	27 (30.7)	26 (20.6)	0.094
Dizziness	36 (16.8)	17 (19.3)	19 (15.1)	0.415
Headache	28 (13.1)	15 (17.0)	13 (10.3)	0.151
Impaired consciousness	16 (7.5)	13 (14.8)	3 (2.4)	< 0.001
Acute cerebrovascular disease	6 (2.8)	5 (5.7)	1 (0.8)	< 0.05
Ataxia	1 (0.5)	1 (1.1)	0 (0.0)	NA
Epilepsy	1 (0.5)	1 (1.1)	0 (0.0)	NA
PNS	19 (8.9)	7 (8.0)	12 (9.5)	0.691
Hypogeusia	12 (5.6)	3 (3.4)	9 (7.1)	0.243
Hyposmia	11 (5.1)	3 (3.4)	8 (6.3)	0.338
Hypoplasia	3 (1.4)	2 (2.3)	1 (0.8)	0.365
Neuralgia	5 (2.3)	4 (4.5)	1 (0.8)	0.074
Muscle injury	23 (10.7)	17 (19.3)	6 (4.8)	< 0.001

PNS = peripheral nervous system; NA = not applicable.

In another clinical series of neurological patients with COVID-19, Helms et al.^66^ described the following neurological disturbances: agitation in 40 of 58 patients (69%), corticospinal tract signs in 39/58 (67%), and dysexecutive syndrome in 14/39 (36%). Brain MRI findings could be summarized as leptomeningeal enhancement in eight of 13 patients (62%), perfusion abnormalities in 11/11 (100%), and ischemic stroke in 3/13 (23%). These data should be interpreted with caution because they did not allow the determination of which of these clinical and radiological characteristics were due to critical illness–related encephalopathy, brain cytokine effects, or the effect of withdrawal of medication, and which features were directly associated with SARS-CoV-2 infection.

Stroke is one of the most frequent neurological diseases associated with SARS-CoV-2 infection,^8^ and large-vessel stroke in younger patients was recently reported in five patients.^67^ The mean National Institutes of Health Stroke Scale score in these patients was 17 (scores range from 0 to 42; the higher the score, the greater the severity of stroke). Intravascular coagulation is the most likely factor in causing ischemic stroke in these patients; a high D-dimer is associated with increased risk for thrombosis and with a poor prognosis in COVID-19 patients.^68,69^

Finally, the presence of SARS-CoV-2 in the human brain was recently documented in the frontal lobe.^70^ A patient with Parkinson’s disease died 11 days after SARS-CoV-2 infection because of cardiac and pulmonary complications. Transmission electron microscopy showed viral particles in frontal lobe sections. Noteworthy, virus-like particles were also found in the capillary endothelium and actively budding across endothelial cells, which could suggest a hematogenous route for SARS-CoV-2 entry into the CNS.

## FUTURE

The real spectrum of neurological manifestations of COVID-19 is an ongoing story. As the virus spreads to all continents, we may observe different manifestations in populations with diverse genetic and environmental backgrounds. Besides, RNA viruses such as SARS-CoV-2 suffer frequent mutations, which can be associated with new and unidentified neurological manifestations. Close epidemiological surveillance is necessary to follow GBS’s frequency and acute demyelinating encephalomyelitis, autoimmune conditions that usually follow viral infections.

Another critical aspect of the COVID-19 pandemic is the collapse of many national health services worldwide. Many neurological diseases require continuous follow-up and regular outpatient visits, and patients with stroke and epilepsy, among other neurological conditions, frequently present to the emergency every day. The impact of the pandemic in the care of patients with other neurological diseases has already been observed in many countries, raising the fear of additional load over an already overburdened health system. Neurological societies are urged to devise guidelines and recommendations based on the best current information available on how neurologists should manage patients with neurological conditions that can be directly affected by COVID-19, such as multiple sclerosis, epilepsy, or myasthenia gravis.

Another exciting field of research is about the long-term neurological consequences of SARS-CoV-2 infection. Because a vast number of people worldwide will be infected, the inflammatory response elicited by SARS-CoV-2 may trigger or accelerate via impaired blood–brain barrier function some subclinical mechanisms that underlie the earliest stages of some neurodegenerative disorders.^71^ Prospective studies on this topic could answer this question in the future.

## CONCLUSION

Although COVID-19 does not appear to have exuberant neurological manifestations like the recent epidemics of the Zika virus, HCoVs have previously demonstrated the potential to invade and damage the nervous system. Therefore, physicians should be alert to new clinical syndromes related to infection and be prepared to face worsening clinical control of preexisting neurological diseases in patients with COVID-19.
